# The beneficial effects of probiotics on patients with coronary heart disease: a systematic review and meta-analysis

**DOI:** 10.3389/fnut.2025.1612021

**Published:** 2025-06-20

**Authors:** Jie Dong, Lei Chen, Nan Zheng, Ming Yang

**Affiliations:** Department of Cardiovascular Surgery, Institute of Cardiac Surgery, The Sixth Medical Center of PLA General Hospital, Beijing, China

**Keywords:** probiotics, coronary heart disease, LDL cholesterol, HDL cholesterol, triglycerides

## Abstract

**Background:**

Coronary heart disease (CHD) is a major global health burden, and emerging evidence suggested that probiotics could improve cardiovascular health by modulating gut microbiota and lipid profiles. However, the efficacy of probiotics remains elusive, indicating the necessity of conducting this meta-analysis.

**Methods:**

This study followed the Preferred Reporting Items for Systematic Reviews and Meta-Analyses (PRISMA) guidelines, searching PubMed, EMBASE, and Web of Science databases for retrieving randomized controlled trials (RCTs) on probiotics’ effects on lipid profiles (low-density lipoprotein (LDL), high-density lipoprotein (HDL), and triglycerides (TG)) in CHD patients. Inclusion and exclusion criteria concentrated on English-language RCTs, and data on study characteristics were extracted. Study quality was assessed using Cochrane and NHLBI tools, and statistical analysis was conducted via R 4.3.2 software.

**Results:**

The literature search identified 263 records, yielding 6 RCTs, 5 of which were included in the meta-analyses. For LDL level (*n* = 278), both fixed-effects and random-effects models exhibited an overall effect size of 1.25 units [95% confidence interval (CI): −0.62 to 3.12] with a low heterogeneity (*I*^2^ = 0%), while probiotics-based monotherapy achieved a mean difference (MD) of 13.4105 (95% CI: −8.0670 to 34.8879) versus an MD of 1.1578 for combination therapy (95% CI: −0.7146 to 3.0302). For HDL level (*n* = 278), the fixed-effects model yielded an MD of −3.8107 (95% CI: −4.2490 to −3.3724) versus an MD of −2.3119 for the random-effects model (95% CI: −4.2290 to −0.3949) with a moderate heterogeneity (*I*^2^ = 61.6%). Combination therapy demonstrated an MD of −2.9848 (95% CI: −4.7965 to −1.1732), while monotherapy exhibited a non-significant MD of 0.9115 (95% CI: −3.5084 to 5.3314). TG analysis yielded a common effect size of 17.95, with a minimal-to-moderate heterogeneity (*I*^2^ = 0 to 84.7%).

**Conclusion:**

Probiotics, particularly monotherapy for LDL and combination therapy for HDL, exhibited potential to improve lipid profiles in CHD patients. However, further research is needed to address existing limitations and confirm efficacy.

## Introduction

Coronary heart disease (CHD) remains a leading cause of morbidity and mortality worldwide, characterized by the accumulation of atherosclerotic plaques in coronary arteries, which is mainly exacerbated by metabolic imbalances, inflammation, and dyslipidemia. This complex multifactorial disease is mainly exacerbated by metabolic imbalances, chronic inflammation, and dyslipidemia—particularly elevated LDL cholesterol, reduced HDL cholesterol, and heightened TG levels. These factors collectively drive CHD progression and its severe clinical manifestations.

Emerging evidence increasingly highlights the critical role of the gut microbiota in overall cardiovascular health. A healthy, balanced gut microbiome contributes to various physiological processes, while dysbiosis, an imbalance in gut microbial composition, has been strongly linked to increased risk factors for CHD. This includes direct impacts on lipid metabolism, inflammation, and even endothelial function, which are all pivotal in atherosclerotic development.

Probiotics, live microorganisms that confer health benefits when administered in adequate amounts, have remarkably garnered researchers’ attention for their potential to modulate gut microbiota, reduce inflammation, improve lipid profiles, and enhance metabolic parameters, thereby providing a promising therapeutic direction for CHD management. Their purported benefits stem from their ability to modulate gut microbiota composition and function, which in turn can lead to a cascade of favorable systemic effects, e.g., reducing systemic inflammation, improving gut barrier integrity, modulating lipid profiles, and enhancing various metabolic parameters that are often deranged in CHD patients. For instance, early research, as evidenced by studies published in 1986 (1), highlighted the association between gut health and cardiovascular risk factors, laying a basis for microbiota’s role in heart disease. Similarly, studies from 1980 (2) and 1985 (3) explored microbial interventions, suggesting preliminary benefits in lipid metabolism and systemic inflammation, which are pivotal in CHD. More recent investigations, including those from 2021 (4) and 2021 (5), have advanced this field by demonstrating the potential of probiotic supplementation to mitigate cardiovascular risk factors through improved gut barrier function and reduced oxidative stress. However, recent research (6) provided insights into the molecular mechanisms underlying probiotics’ effects on lipid profiles and endothelial function in CHD patients.

Despite these promising findings, the therapeutic value of probiotics in CHD remains inconclusive due to variability in study designs, intervention protocols, patient populations, and outcome measures. This inconsistency highlights the need for a comprehensive synthesis of existing evidence to clarify the efficacy and safety of probiotics as an adjunctive or standalone intervention for CHD.

Therefore, this meta-analysis and systematic review aimed to rigorously evaluate the beneficial effects of probiotics, either as monotherapy or in combination with other agents, on key cardiovascular biomarkers, including LDL, HDL, and TG levels, in patients with CHD. By synthesizing data from randomized controlled trials (RCTs), a robust, evidence-based assessment of probiotics’ impact was presented, along with identifying potential sources of heterogeneity, thereby providing insights into optimal intervention strategies and guiding clinical practice and future research directions in the management of CHD. This approach has addressed the gaps in current literature, utilizing a systematic methodology to enhance the reliability and generalizability of findings across diverse populations and settings.

## Methods

### Literature search

This meta-analysis was conducted in accordance with the Preferred Reporting Items for Systematic Reviews and Meta-Analyses (PRISMA) guidelines. The literature search was performed across PubMed, EMBASE, and Web of Science databases from their inception up to a specified cutoff date. The search strategy employed a combination of Medical Subject Headings (MeSH terms) and free-text keywords to maximize sensitivity. Specific search terms included, but were not limited to: “Probiotics” [MeSH Terms] OR “Probiotic*” [Free Text] AND “Coronary Heart Disease” [MeSH Terms] OR “Coronary Artery Disease” [Free Text] OR “Myocardial Ischemia” [Free Text] AND “Randomized Controlled Trial” [Publication Type] OR “RCT” [Free Text].

### Inclusion criteria

Studies included in this meta-analysis were required to meet the following criteria: they were RCTs published fully in English, involved patients diagnosed with CHD or related cardiovascular conditions, and evaluated interventions involving probiotics either alone or in combination with other agents, compared to a control group, typically placebo or an alternative treatment.

### Exclusion criteria

Exclusion criteria encompassed ongoing studies, duplicate publications, reviews, meta-analyses, case reports, conference abstracts, and studies involving populations with unrelated conditions, such as type 1 diabetes or non-cardiovascular diseases.

### Data extraction and outcome measures

Data extraction was systematically performed to capture key study characteristics, including the first author, publication year, country of origin, RCT registration number, disease profile of participants, study period, age range, intervention type, and primary outcomes. For intervention type, detailed information was extracted regarding the nature of probiotic administration. This included whether probiotics were administered as monotherapy or in combination with other agents. For studies involving combination therapy, we specifically noted the type of co-intervention, such as but not limited to: (1) Nutritional supplements: (e.g., vitamins, minerals, fiber, other dietary compounds); (2) Pharmacological agents: (e.g., statins, anti-inflammatory drugs); (3) Lifestyle interventions: (e.g., dietary advice, exercise programs). This granular extraction allowed for subsequent subgroup analyses to differentiate the effects of probiotics alone versus when combined with other active treatments, and to understand the specific context of these combinations. Additional quality-related information was also recorded.

### Quality assessment of individual studies

The quality of individual studies was assessed by two independent investigators using the Cochrane Collaboration’s risk of bias tool, supplemented by the NHLBI’s Quality Assessment of Controlled Intervention Studies tool, evaluating domains, such as random sequence generation, allocation concealment, participants’ and personnels’ blinding, blinding of outcome assessment, incomplete outcome data, selective reporting, and other potential biases. Each domain was rated as low, high, or unclear risk, and an overall quality score was determined for each study.

### Statistical analysis

For statistical analysis, continuous outcomes were assessed by calculating mean differences (MDs) with 95% confidence intervals (CIs). The MDs were calculated as the mean change in the control group minus the mean change in the probiotic group (Control group – Probiotic group). A Fixed-effects model was used when heterogeneity, evaluated via the I^2^ statistic, was below 50%, while a random-effects model was employed when I^2^ exceeded 50%. Heterogeneity was further quantified using tau^2^ and tested with the Q statistic. Subgroup analysis was performed based on intervention type (probiotics-based monotherapy vs. combination therapy) to explore potential differences in effect sizes. Statistical significance was defined as a *p*-value of less than 0.05 for two-tailed tests. All analyses were conducted using appropriate statistical software packages, and figures were generated to illustrate the study selection process, risk of bias, and meta-analytic results. R 4.3.2 software was utilized for analysis and visualization (7).

## Results

### Literature search results

**Figure 1:**
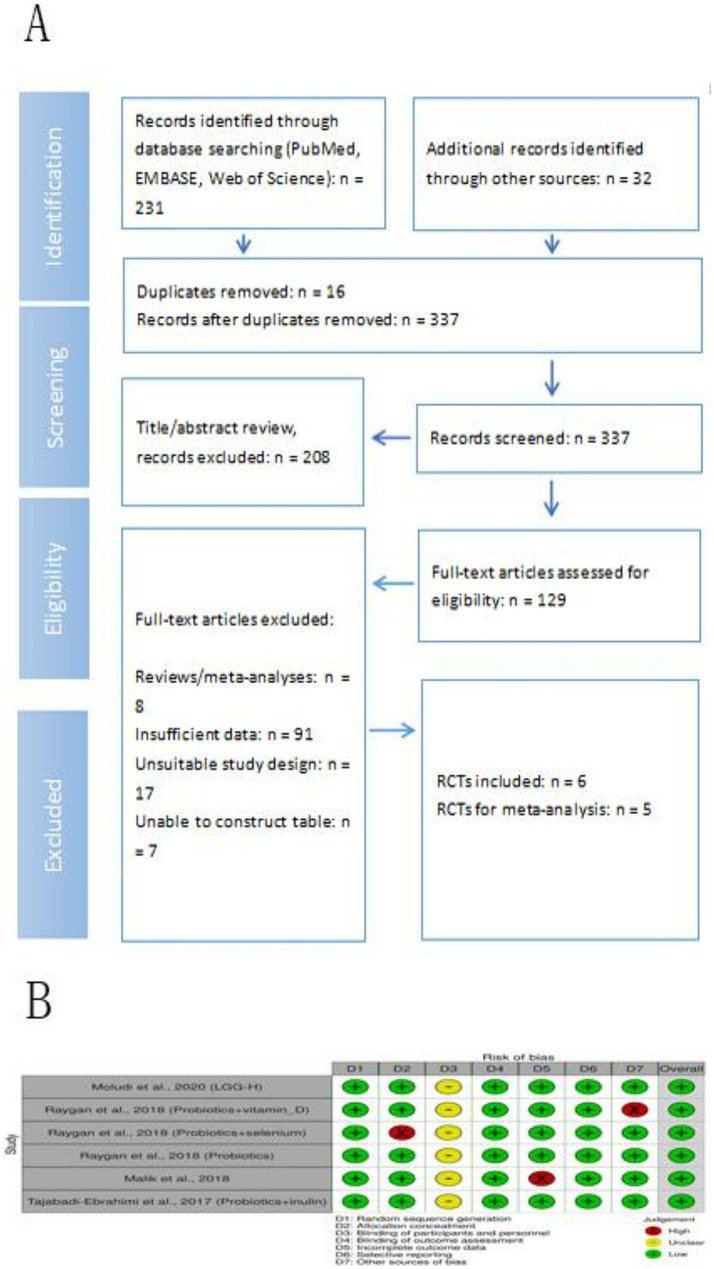
Study selection and risk of bias assessment. **(A)** Flow diagram of study selection process. **(B)** Risk of bias graph.

The literature search identified a total of 263 records, comprising 231 from database searches across PubMed, EMBASE, and Web of Science, and an additional 32 from other sources. After removing 16 duplicates, 247 unique records remained for screening. Following the title and abstract review, 208 records were excluded, leaving 129 full-text articles assessed for eligibility. Of these, 123 were excluded for reasons such as being reviews or meta-analyses (*n* = 8), lacking insufficient data (*n* = 91), employing inappropriate study designs (*n* = 17), or being unable to construct tables (*n* = 7). Ultimately, 6 RCTs were involved in the study, in which 5 of these RCTs were regarded appropriate for meta-analysis (Figure 1A).

The risk of bias assessment across the six included studies indicated generally low risk in random sequence generation, allocation concealment, blinding of outcome assessment, incomplete outcome data, selective reporting, and other sources of bias. However, some variability was noteworthy, including unclear blinding of participants and personnel in all studies, a high risk of allocation concealment in one study, and a high risk of incomplete outcome data and other bias in specific studies (Figure 1B).

### Characteristics of included studies

The included studies, spanning from 2017 to 2020, consisted of RCTs (8–13) that were conducted primarily in Iran, with one study from the USA, concentrating on patients with cardiovascular diseases, such as type 2 diabetes with CHD, overweight type 2 diabetic patients with CHD, stable coronary artery disease, and general coronary artery diseases. These studies, registered under various RCT numbers (e.g., IRCT201503025623N37 and NCT01952834), were conducted between 2013 and 2018. Participants age ranged from 40 to 85 years, although one study reported a mean age range of 51.19–53.96 years. The evaluated interventions included probiotics, vitamin D combined with probiotics, and selenium combined with probiotics. Specifically, studies utilized various probiotic formulations; for example, Malik et al., 2018, investigated the effects of *Lactobacillus plantarum* 299v (Lp299v). Other studies employed multi-strain probiotic supplements or synbiotics (probiotics combined with prebiotics). Primary outcomes assessed across these trials were diverse, encompassing insulin metabolism parameters, mental health and metabolic status, HOMA-IR (insulin resistance), vascular endothelial function, and anthropometric indices, alongside lipid profiles, reflecting a broad interest in metabolic, cardiovascular, and psychological endpoints among these patient populations. The quality assessment, conducted using the NHLBI’s Quality Assessment of Controlled Intervention Studies tool, rated all six studies as fair (8–13) (Table 1).

**Table 1:** Characteristics of included studies.

Study	Country	Study design	RCT Registration Number	Disease type	Study period	Age range (years)	Intervention type	Primary outcome	Study quality
Tajabadi-Ebrahimi et al., 2017 (8)	Iran	RCT	IRCT201503025623N37	Overweight type 2 diabetic patients with coronary heart disease	Mar-Jun 2015	40–85	Synbiotic vs. placebo	Insulin metabolism parameters	Fair
Raygan et al., 2018 (Vitamin D + Probiotic) (9)	Iran	RCT	IRCT2017073033941N4	Type 2 diabetic patients with coronary heart disease	Aug-Nov 2017	45–85	Vitamin D + Probiotic vs. Placebo	Mental health and metabolic status	Fair
Raygan et al., 2018 (Probiotic) (10)	Iran	RCT	IRCT2017082733941N5	Type 2 diabetic patients with coronary heart disease	Oct 2017-Jan 2018	40–85	Probiotic vs. placebo	Insulin metabolism	Fair
Raygan et al., 2018 (Selenium + Probiotic) (11)	Iran	RCT	IRCT20170513033941N28	Type 2 diabetic patients with coronary heart disease	Dec 2017-Mar 2018	45–85	Selenium + Probiotic vs. Placebo	HOMA-IR	Fair
Malik et al., 2018 (12)	USA	RCT	NCT01952834	Stable coronary artery disease	2013–2015	40–75	Lp299v vs. Vancomycin	Vascular endothelial function	Fair
Moludi et al., 2020 (13)	Iran	RCT	IRCT20121028011288N15	Coronary artery diseases	Jul-Oct 2018	Mean: 51.19–53.96	Probiotic vs. Placebo	Anthropometric indices, lipid profile	Fair

### The beneficial effects of probiotics on LDL levels in patients with CHD

**Figure 2:**
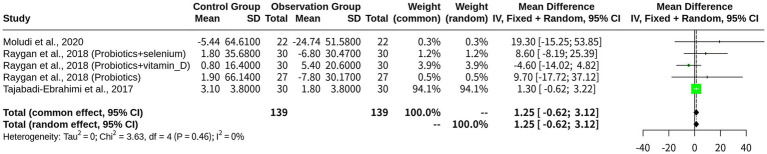
Meta-analysis of LDL outcomes across included studies.

A total of five studies (*k* = 5) comprising 278 observations were included in the LDL analysis. The results revealed that both the fixed-effects model and the random-effects model yielded similar estimates of the overall effect size, which was approximately 1.25 units (95% CI: −0.62 to 3.12). However, the heterogeneity test revealed a low I^2^ value of 0%, indicating a high level of consistency in the effect sizes between studies. This suggests that the fixed-effects model appeared more appropriate for this analysis, which assumes a common effect size among all studies and assigns more weight to studies with larger sample sizes. The quantification of heterogeneity further supported this conclusion, yielding a tau^2 value of 0, indicating no between-study variance. The heterogeneity test also revealed a low Q statistic (3.63) and *p*-value (0.4579), indicating that the studies were not significantly heterogeneous (Figure 2).

**Figure 3:**
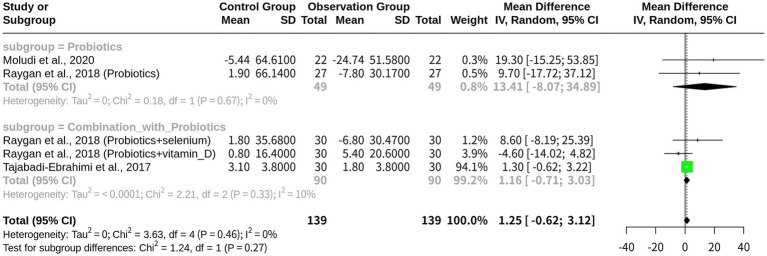
Subgroup meta-analysis on LDL by intervention type: probiotics-based monotherapy vs. combination therapy.

The probiotics-based monotherapy subgroup included two studies, reporting an MD of 13.4105, suggesting a substantial effect. However, the wide 95% CI (−8.0670 to 34.8879) indicated high uncertainty in the estimate. The “Combination with Probiotics” subgroup consisted of three studies, with an MD of 1.1578 and a 95% CI of −0.7146 to 3.0302, highlighting a smaller effect. Heterogeneity in each subgroup was low (*I*^2^ = 0.0% for monotherapy and *I*^2^ = 9.5% for combination therapy), indicating relative consistency among studies. The test for subgroup differences revealed no significant difference between the two subgroups (*Q* = 1.24, d.f. = 1, *p*-value = 0.2653) (Figure 3).

### The beneficial effects of probiotics on HDL levels in patients with CHD

**Figure 4:**
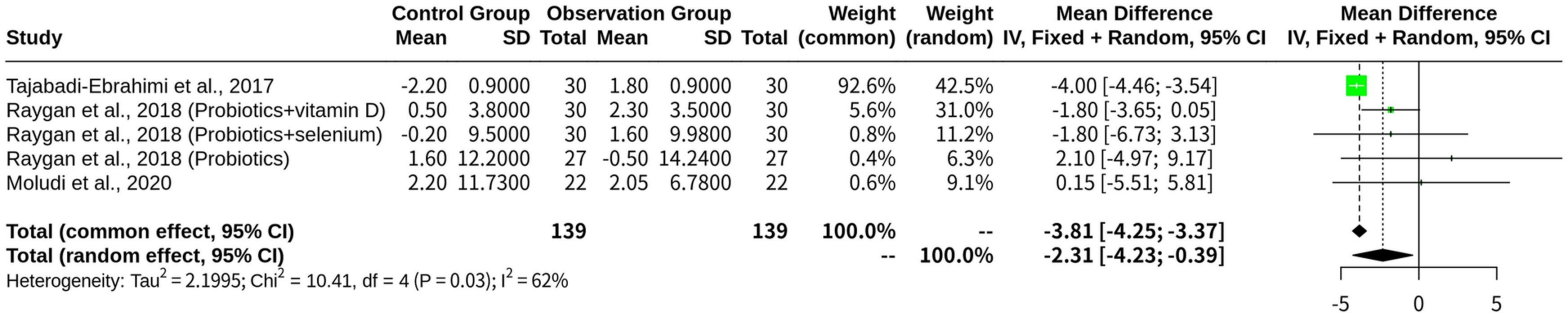
Meta-analysis of HDL outcomes across included studies.

With 5 studies included and a total of 278 observations, the results indicated that probiotics exhibited to have a significant effect on increasing HDL level. The fixed-effects model revealed a largely negative MD of −3.8107, indicating a substantial increase in HDL level, with a corresponding 95% CI of [−4.2490; −3.3724]. In contrast, the random-effects model yielded a smaller and less precise MD of −2.3119, with a wider CI of [−4.2290; −0.3949], although being statistically significant at *p* = 0.0181. The heterogeneity analysis revealed an I^2^ value of 61.6%, indicating moderate-to-high heterogeneity among the studies. Further analysis using the Q-profile method confirmed this finding, with a significant heterogeneity test result (*p*-value = 0.0341) (Figure 4).

**Figure 5:**
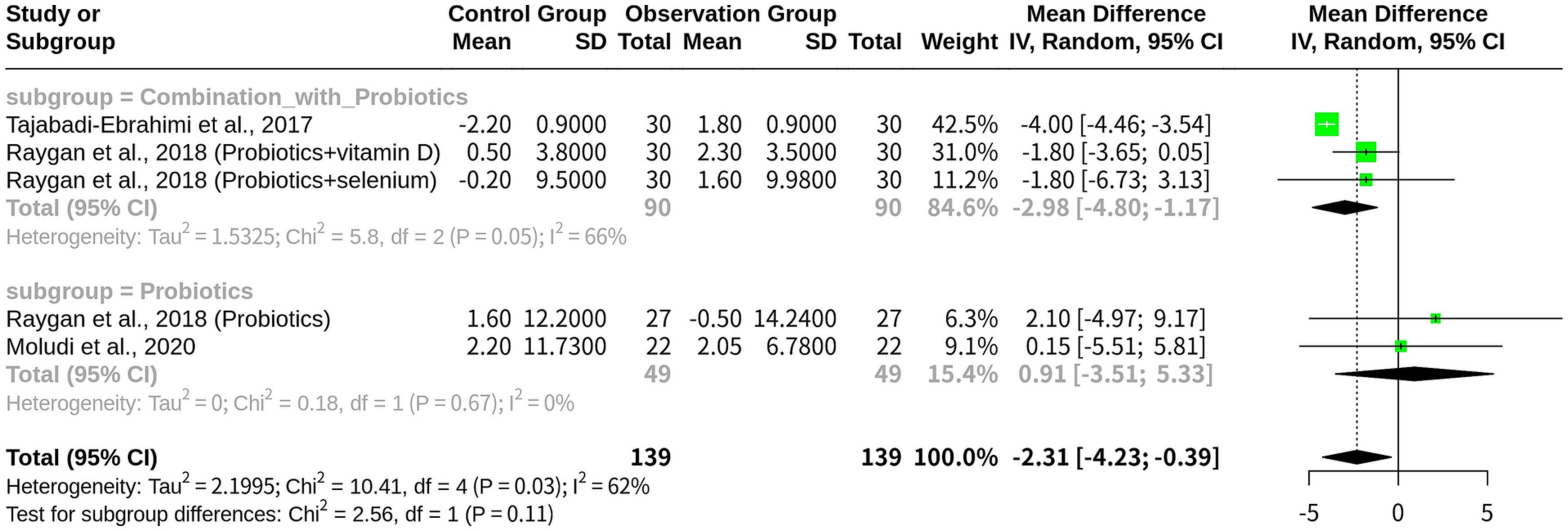
Subgroup meta-analysis on HDL by intervention type: probiotics-based monotherapy vs. combination therapy.

Subgroup analysis was performed using a random-effects model based on the intervention type: ‘Combination with Probiotics’ (*k* = 3) and ‘Probiotics monotherapy’ (*k* = 2). The subgroup “Combination with Probiotics” exhibited a significant effect size (MD = -2.9848), indicating an increase in HDL level compared with the control group, with a 95% CI of (−4.7965; −1.1732) and a tau^2^ value of 1.5325, suggesting moderate heterogeneity in this subgroup. In contrast, the “Probiotics” subgroup showed no significant effect (MD = 0.9115), with a wide 95% CI of (−3.5084; 5.3314) and a tau^2^ value of 0, indicating no heterogeneity in this subgroup. The test for subgroup differences resulted in a non-significant *p*-value of 0.1099, suggesting that the differences between these two subgroups were not statistically significant. Additional results included z-values (−2.36), *p*-values (0.0181 and 0.0341), tau^2^ values (1.4831 and 2.1995), *I*^2^ values (61.6 and 65.5%), *Q* values (10.41 and 2.56), and degrees of freedom (4 and 1) (Figure 5).

### The beneficial effects of probiotics on TG levels in patients with CHD

**Figure 6:**
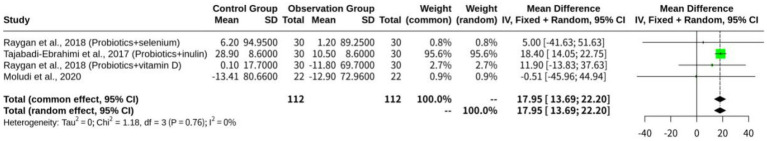
Meta-analysis of TG outcomes across included studies.

The results of the beneficial effects of probiotics on patients with CHD, specifically regarding their TG levels indicated that both models yielded similar estimates of the common effect size, which was approximately 17.95, indicating a significant reduction in TG levels following probiotic treatment. However, despite the similar effect sizes, the I^2^ value indicated minimal-to-moderate heterogeneity among the studies, ranging from 0 to 84.7%, suggesting that either model could be appropriate for this analysis. The heterogeneity analysis also supported this conclusion, with a *p*-value of 0.7570 and a Chi-square value of 1.18 on three degrees of freedom (Figure 6).

### Probiotics’ impact on inflammation and other cardiovascular markers

Beyond their primary impact on lipid profiles, probiotics and their co-interventions have also shown effects on other critical cardiovascular risk markers, particularly inflammation. Several studies in this meta-analysis reported changes in high-sensitivity C-reactive protein (hs-CRP), a key inflammatory biomarker associated with CHD progression. For instance, Raygan et al. (9) (Vitamin D + Probiotic), observed a significant reduction in serum hs-CRP (−950.0 ± 1811.2 vs. +260.5 ± 2298.2 ng/mL, *p* = 0.02) with vitamin D and probiotic co-supplementation. Similarly, probiotic monotherapy in another study by Raygan et al. (10) (Probiotic), led to a significant reduction in serum hs-CRP (*β* − 0.88 mg/L; 95% CI − 1.39, −0.38; *p* = 0.001). The combination of selenium and probiotics also demonstrated a notable decrease in hs-CRP (β- 1043.28 ng/mL; 95% CI, − 1929.67, −156.89; *p* = 0.02) in the study by Raygan et al. (11) (Selenium + Probiotic). These consistent reductions in hs-CRP across various probiotic and co-intervention strategies suggest a beneficial anti-inflammatory role that may contribute to improved cardiovascular outcomes, complementing the observed lipid-modulating effects. However, it’s important to note that while some studies, such as Malik et al. (12), reported a modest elevation in blood pressure post-Lp299v supplementation in their specific cohort, this finding conflicted with prior research and was potentially influenced by significant differences in patient populations, including smoking status, underscoring the complexity of these secondary outcomes and the need for further focused research on these specific markers in diverse CHD populations.

## Discussion

The present meta-analysis on LDL outcomes in patients with CHD provided valuable insights into the comparative efficacy of probiotics as monotherapy versus combination therapy. The findings highlight several important points for discussion, including clinical implications, heterogeneity, and potential mechanisms. The results unveiled a significant difference in MD between monotherapy and combination therapy on LDL level, in which monotherapy demonstrated a notably larger MD (13.4105) compared with combination therapy (1.1578). This suggests that probiotics alone may exert a more remarkable effect on reducing LDL level in CHD patients, although the wide 95% CI (−8.0670 to 34.8879) for monotherapy indicates notable uncertainty and potential variability in clinical outcomes. In contrast, the narrower CI (−0.7146 to 3.0302) for combination therapy reflects greater precision, while a more modest effect size is noteworthy. Clinically, this discrepancy may demonstrate that probiotics alone may be more effective for specific CHD patient subgroups with hyperlipidemia, while combination therapies (e.g., with vitamin D or selenium supplementation) may provide broader, but less potent lipid-lowering effects, possibly due to interactions or dilution of probiotic effects by co-interventions. Evidence suggests that specific probiotic strains, such as *Lactobacillus* and *Bifidobacterium*, can significantly lower LDL level by modulating bile acid metabolism and cholesterol absorption, as supported by systematic reviews and meta-analyses (14). Beyond these well-established pathways, probiotics may also influence LDL levels through other mechanisms. For instance, some strains can produce short-chain fatty acids (SCFAs) like propionate, which can inhibit cholesterol synthesis in the liver. Additionally, probiotics can reduce systemic inflammation and oxidative stress, both of which are known to contribute to dyslipidemia and plaque formation. By strengthening the gut barrier, they can decrease the translocation of lipopolysaccharides (LPS) from the gut lumen into circulation, thereby reducing chronic low-grade inflammation that negatively impacts lipid metabolism. The indirect effects of vitamin D supplementation on lipid metabolism, potentially interacting with probiotics, may diminish LDL-lowering outcomes, while selenium’s role in antioxidant and immune regulation may have a less direct impact on LDL reduction (15).

Both subgroups demonstrated low heterogeneity (I^2^ = 0.0% for monotherapy, 9.5% for combination therapy), indicating consistency in each group. However, the lack of significant subgroup differences (*Q* = 1.24, *p* = 0.2653) suggests that the intervention type (monotherapy vs. combination therapy) does not significantly account for the variability observed in LDL outcomes. This consistency could reflect similar study designs, patient populations, or probiotic strains across trials, while it also raises questions about whether other unmeasured factors, such as baseline LDL level, dietary habits, or gut microbiota profiles, may influence outcomes. The role of gut microbiota in cholesterol metabolism provides a mechanistic basis for the more significant LDL-lowering effect of monotherapy, as individual differences in microbiota composition may contribute to variability in therapeutic responses (16, 17).

The meta-analysis on HDL outcomes in patients with CHD provided critical insights into the comparative efficacy of probiotics as monotherapy versus combination therapy, involving significant differences from the LDL outcomes. The analysis revealed a significantly negative MD in HDL level with probiotics, indicating an increase in HDL level, particularly in the combination therapy subgroup (MD = −2.9848, 95% CI: −4.7965 to −1.1732) compared with probiotics-based monotherapy (MD = 0.9115, 95% CI: −3.5084 to 5.3314), which showed no significant effect. This suggests that combination therapies (e.g., probiotics with vitamin D or selenium supplementation) may be more effective in elevating HDL level in CHD patients, potentially providing greater cardiovascular protection by improving the lipid profile. Clinically, this contrasts with LDL results, where probiotics-based monotherapy exhibited a larger effect size (MD = 13.4105) compared with combination therapy (MD = 1.1578), indicating a stronger LDL-lowering effect for monotherapy. The difference could reflect probiotics’ distinct mechanisms on HDL level versus LDL level, in which combination therapies could increase HDL level through synergistic anti-inflammatory or metabolic effects, such as those driven by vitamin D or selenium’s immunomodulatory properties. A meta-analysis of probiotics’ effects on lipid profiles also confirmed that combination therapies may more effectively raise HDL level through enhanced anti-inflammatory actions and metabolic synergy (18).

In contrast to the low heterogeneity observed in LDL outcomes (*I*^2^ = 0%), HDL outcomes exhibited moderate-to-high heterogeneity [*I*^2^ = 61.6% (overall), *I*^2^ = 65.5% (in subgroups)], suggesting variability across studies in HDL outcomes. The random effects model was applied to HDL due to the observed heterogeneity, in contrast to the fixed-effects model, which was applicable for LDL (*I*^2^ = 0%). In the subgroups, combination therapy demonstrated moderate heterogeneity (tau^2^ = 1.5325), while monotherapy exhibited no heterogeneity (tau^2^ = 0), suggesting consistent effects in each group. However, overall variability might be attributed to differences in probiotic strains, patient demographics, or co-interventions. The heterogeneity in HDL outcomes, absent in LDL, could be associated with HDL’s sensitivity to environmental factors, such as diet or inflammation, which vary across studies and could be influenced by unmeasured confounding factors, involving gut microbiota composition, dietary habits, and inflammation level (19). HDL level is particularly susceptible to these factors, contributing to the observed variability compared with LDL level’s stable response.

The significant increase in HDL level with combination therapy might result from enhanced anti-inflammatory effects or metabolic synergy between probiotics and co-interventions (e.g., vitamin D or selenium supplementation), potentially upregulating HDL synthesis or improving reverse cholesterol transport. In contrast, probiotics alone might not sufficiently modulate HDL pathways, as indicated by the non-significant effect in monotherapy. This could be attributed to a limited impact on HDL-specific mechanisms, such as apolipoprotein A-I production or cholesterol efflux, differing from LDL-lowering pathways, such as bile acid metabolism and cholesterol assimilation. This mechanistic distinction explains why LDL level exhibited a stronger response to monotherapy, whereas HDL benefits more from combination therapy, highlighting the involvement of distinct lipid modulation pathways. Probiotics may influence HDL level by regulating apolipoprotein A-I production and cholesterol efflux, and combination therapies potentially enhance these effects through synergistic mechanisms (20). Specifically, probiotics may enhance HDL levels by promoting the expression of genes involved in reverse cholesterol transport, such as ABCA1 and ABCG1, which facilitate cholesterol efflux from peripheral cells to HDL particles. They might also modulate the activity of enzymes critical for HDL metabolism, such as lecithin-cholesterol acyltransferase (LCAT), which is crucial for HDL maturation. Furthermore, the anti-inflammatory effects of certain probiotic strains could indirectly support HDL function, as inflammation is known to impair HDL’s antioxidant and anti-inflammatory properties.

Differences in HDL outcomes could arise from variations in patients’ characteristics, such as baseline HDL level, CHD severity, or genetic predispositions, interacting differently with probiotics alone versus in combination. For instance, combination therapies could be more beneficial for patients with complex metabolic profiles, while monotherapy could be advantageous for those with isolated hyperlipidemia. No similar pattern was found for LDL, where monotherapy consistently exhibited greater efficacy. Study design-associated factors, such as probiotic strain selection, dosage, and duration, could also differentially influence HDL and LDL levels, in which HDL appeared more responsive to co-interventions or environmental factors. The significant increase in HDL level with combination therapy suggests that it may be a preferred approach for CHD patients seeking to improve HDL levels and overall lipid profiles, providing a dual benefit alongside the stronger LDL reduction observed with monotherapy. However, the non-significant effect of monotherapy on HDL level and its wide CI indicate caution in recommending it solely for HDL elevation, unlike LDL where monotherapy was more promising. Future research should explore strain-specific effects, optimal combinations, and long-term HDL outcomes, as well as investigating dietary, genetic, and lifestyle factors influencing HDL responses, contrasting these with LDL findings to tailor interventions. Larger trials are needed to reduce uncertainty in HDL estimates and validate subgroup differences.

The moderate-to-high heterogeneity in HDL outcomes, being absent in LDL outcomes, warrants further investigation into unmeasured confounders, such as gut microbiota composition, dietary patterns, or inflammation levels, disproportionately influencing HDL responses. The small number of studies involved could limit the generalizability of HDL findings, whereas LDL’s low variability suggests more consistent effects. Additional research is needed to better understand these differences and validate the observed patterns. Publication bias and study quality should also be assessed to ensure robust conclusions.

### Limitations

This meta-analysis, while providing valuable insights into the effects of probiotics on lipid profiles in patients with CHD, has several limitations that warrant consideration. Firstly, the relatively small number of included studies (*n* = 5 for both LDL and HDL analyses) could restrict the generalizability of the findings and increase the risk of publication bias, as the number of studies was insufficient to formally assess publication bias using statistical tests (e.g., funnel plots or Egger’s test). Secondly, the heterogeneity observed in HDL outcomes (*I*^2^ = 61.6%), rather than in LDL outcomes (*I*^2^ = 0%), could be indicative of potential unmeasured confounders, such as dietary patterns, lifestyle factors, or genetic variations among participants, which were not consistently reported across studies and could influence the observed effects. Thirdly, the variability in probiotic strains, dosages, durations, and co-interventions (e.g., vitamin D and selenium supplementation) across trials could introduce uncertainty, as these factors might differentially impact LDL and HDL levels, while the data lacked sufficient detail to conduct comprehensive subgroup analyses beyond intervention type. Fourthly, the study populations were predominantly from Iran with one from the USA, potentially limiting the applicability of findings to other ethnic or geographical groups with different gut microbiota profiles or CHD risk factors. Finally, the quality of the included studies, rated as fair by the NHLBI’s Quality Assessment of Controlled Intervention Studies tool, indicates potential biases (e.g., unclear blinding of participants and personnel in all studies), influencing the reliability of the results. These limitations highlight the need for larger, more diverse, and rigorously designed trials to confirm and extend these findings.

## Conclusion

This meta-analysis demonstrated that probiotics, either as monotherapy or combination therapy, could be advantageous for improving lipid profiles in patients with CHD, accompanying by distinct effects on LDL and HDL levels. Probiotics-based monotherapy exerted a more remarkable effect on reducing LDL level, suggesting its potential as a targeted intervention for CHD patients with hyperlipidemia, despite remarkable uncertainty indicated by a wide CI. In contrast, combination therapies, particularly with vitamin D or selenium supplementation, exhibited to be more effective in increasing HDL level, providing greater cardiovascular protection by enhancing the lipid profile, although with moderate heterogeneity across studies. The lack of significant subgroup differences between monotherapy and combination therapy for both LDL and HDL outcomes underscores the need for further research to elucidate optimal intervention strategies, including strain-specific effects, dosages, and durations, as well as the role of confounding factors, such as diet and gut microbiota composition. These findings support the therapeutic potential of probiotics in CHD management, while larger, more homogeneous trials are required to address the identified limitations, reduce uncertainty, and develop clinical guidelines for integrating probiotics into routine cardiovascular care.

## Data Availability

The original contributions presented in the study are included in the article/supplementary material, further inquiries can be directed to the corresponding author.
